# Tropical forest light regimes in a human‐modified landscape

**DOI:** 10.1002/ecs2.2002

**Published:** 2017-11-22

**Authors:** Sophie Fauset, Manuel U. Gloor, Marcos P. M. Aidar, Helber C. Freitas, Nikolaos M. Fyllas, Mauro A. Marabesi, André L. C. Rochelle, Alexander Shenkin, Simone A. Vieira, Carlos A. Joly

**Affiliations:** ^1^ School of Geography University of Leeds Leeds LS2 9JT UK; ^2^ Departamento de Biologia Vegetal Instituto de Biologia Universidade Estadual de Campinas Rua Monteiro Lobato, Cidade Universitâria Campinas Sao Paulo 13083‐862 Brazil; ^3^ Instituto de Botânica de São Paulo Avenida Miguel Stéfano Sao Paulo 04301‐902 Brazil; ^4^ Departamento de Física Faculdade de Ciências Universidade Estadual Paulista Avenida Engenheiro Luiz Edmundo Carrijo Coube, 14‐01 Bauru Sao Paulo 17033‐360 Brazil; ^5^ Centro de Meteorologia – IPMet/UNESP Estrada Municipal José Sandrin Bauru Sao Paulo 17048‐699 Brazil; ^6^ Environmental Change Institute School of Geography and the Environment University of Oxford South Parks Road Oxford OX1 3QY UK; ^7^ Núcleo de Estudos e Pesquisas Ambientais Universidade Estadual de Campinas Rua dos Flamboyants, 155 Campinas Sao Paulo 13083‐867 Brazil

**Keywords:** Atlantic forest, Brazil, canopy, degraded, disturbance, extinction coefficient, fragment, montane, radiation, secondary, structure, vertical profile

## Abstract

Light is the key energy input for all vegetated systems. Forest light regimes are complex, with the vertical pattern of light within canopies influenced by forest structure. Human disturbances in tropical forests impact forest structure and hence may influence the light environment and thus competitiveness of different trees. In this study, we measured vertical diffuse light profiles along a gradient of anthropogenic disturbance, sampling intact, logged, secondary, and fragmented sites in the biodiversity hot spot of the Atlantic forest, southeast Brazil, using photosynthetically active radiation sensors and a novel approach with estimations of vertical light profiles from hemispherical photographs. Our results show clear differences in vertical light profiles with disturbance: Fragmented forests are characterized by rapid light extinction within their low canopies, while the profiles in logged forests show high heterogeneity and high light in the mid‐canopy despite decades of recovery. The secondary forest showed similar light profiles to intact forest, but with a lower canopy height. We also show that in some cases the upper canopy layer and heavy liana infestations can severely limit light penetration. Light extinction with height above the ground and depth below the canopy top was highest in fragmented forest and negatively correlated with canopy height. The novel, inexpensive, and rapid methods described here can be applied to other sites to quantify rarely measured vertical light profiles.

## Introduction

Light is a key environmental variable driving plant productivity by providing energy for photosynthesis. Photosynthetic rates respond to changes in incoming radiation, and plants also show acclimation to the prevailing growth irradiance, with lower rates of photosynthesis and respiration and altered leaf structure under shaded conditions (Chen et al. 2014). In tropical forests, which constitute a globally significant store of carbon (Pan et al. 2011) and biodiversity (Dirzo and Raven 2003), the light environment is highly complex (Chazdon and Fetcher 1984) and tree species life histories are often associated with light availability (Poorter et al. 2006). Diurnal and seasonal patterns of incoming radiation can be easily understood based on solar geometry and the movements of clouds. However, the attenuation of light within the forest canopy is determined by the vertical structure of stems, leaves, and their optical properties (Binkley et al. 2013). Hence, forest structure is a driver of light availability within canopies, which can then impact the physiological rates of trees.

Anthropogenic disturbances, such as selective logging, clear felling, fragmentation, and fire, affect forest structure. For example, secondary, logged, fragmented forests, and forest edges typically contain fewer large trees than intact forest (Laurance et al. 1997, Paula et al. 2011, Berenguer et al. 2014). The structure of trees themselves may also be different in disturbed forests due to changes in species composition with the proliferation of pioneers (Michalski et al. 2007, Laurance et al. 2011, Paula et al. 2011). Early successional species have different architectures to shade‐tolerant species with narrower crowns and taller heights (Montgomery and Chazdon 2001, Poorter et al. 2006), and tree architecture has been shown to change after selective logging with lower tree heights for a given diameter (Rutishauser et al. 2016). Hence, light regimes in human‐modified forests may differ from those in intact forests due to differences in tree size‐class distributions, species composition, and allometry.

The majority of research conducted thus far into light environments in human‐modified forests focuses on the understory due to the logistical challenges of working in the canopy. Even with differing structures, the percentage of light reaching the forest floor (transmittance, *T*) is typically low (1–2%) and may vary little between intact, secondary, and selectively logged forest (Nicotra et al. 1999, Montgomery and Chazdon 2001). However, others have shown higher understory light levels in selectively logged forests (Yamada et al. 2014, Osazuwa‐Peters et al. 2015) and decreasing light with secondary forest age (Denslow and Guzman 2000, Lebrija‐Trejos et al. 2011). Spatial heterogeneity in understory light transmittance between gap and closed areas within intact forest has been quantified (Chazdon and Fetcher 1984, Canham et al. 1990, Rich et al. 1993) and shows less variation in secondary forests (Nicotra et al. 1999). Fragment edges are known to have brighter, hotter microclimates compared with forest interiors (Newmark 2001, Ewers and Banks‐Leite 2013, Magnago et al. 2015), although time and structure development since fragmentation, and land‐use adjacent to the fragment, affect the strength of the difference (Didham and Lawton 1999).

Much less information is available on the vertical profile of light, especially in human‐modified forests. The vertical pattern of light transmission is important for our understanding of forest productivity, growth, and dynamics (as inferred from light detection and ranging [LiDAR] data, Stark et al. 2012, 2015). A small number of datasets have been collected for intact tropical forests with direct measurements of light profiles (Yoda 1974, Torquebiau 1988, Maass et al. 1995, Anhuf and Rollenbeck 2001, Wirth et al. 2001, Parker et al. 2005) or of leaf area profiles (Clark et al. 2008). However, most studies are limited to very small sample numbers (but see Parker et al. 2005, Clark et al. 2008) or to a small footprint from crane‐based studies (Anhuf and Rollenbeck 2001, Kitajima et al. 2005). Data on 3D forest structure (Lefsky et al. 2002) and associated light environments (Parker et al. 2001, Stark et al. 2012) can be estimated from LiDAR, which increases the spatial coverage of measurements. However, LiDAR technology remains expensive and produces vast datasets that can be challenging to analyze. Diffuse light conditions (as opposed to direct light conditions) are convenient for the direct measurement of vertical light profiles because it avoids the high variability in light conditions due to sunflecks (e.g., Parker et al. 2002 found profiles measured under an overcast sky were smoother than those under clear sky) and the profiles produced reflect the underlying forest structure. Further, diffuse light can penetrate deeper into forest canopies than direct light resulting in more efficient canopy light use under diffuse light (Alton et al. 2007). Therefore, here we focus on diffuse light.

With this paper, we tackle the data gap concerning vertical light profiles for intact and human‐modified Atlantic forests. This work will add to previous knowledge of understory light patterns in intact and human‐modified forests and provide valuable new data for a threatened biodiversity hot spot, which has experienced substantial deforestation (Ribeiro et al. 2009). As datasets on vertical light profiles are rare, especially in disturbed forests, the results will be of use to test light interception schemes for forest models. We use two methods to measure light profiles—directly with photosynthetically active radiation (PAR) sensors and indirectly with vertical profiles of hemispherical photographs. Both methods are low cost and repeatable across landscapes. The aim of the paper was to characterize the vertical light environments of forests along a disturbance gradient of intact, selectively logged, secondary, and fragmented forest, accounting for spatial variation within sites. We expect that along the gradient from least to most disturbed forests (intact < logged < secondary < fragment), light will penetrate further into the canopy due to lower canopy closure and smaller tree crowns with increasing severity of disturbance.

## Materials and Methods

### Study sites

The study was carried out in and around Núcleo Santa Virginia of the Serra do Mar State Park in the municipality of São Luis do Paraitinga, São Paulo state, Brazil. The park is home to the largest contiguous patch of Atlantic forest remaining, running along a steep coastal mountain range. The forest is classified as montane moist dense forest (Veloso et al. 1991, Oliveira‐Filho and Fontes 2000) and contains palms, tree ferns, bamboos, epiphytes, and lianas in addition to dicot trees. Mean annual precipitation is 2300 mm with a dry season in July and August, mean annual temperature is 17°C (Joly et al. 2012), and fog occurs frequently (Rosado et al. 2010). Inland from the park, the landscape is pastoral with patches of privately owned forest within a matrix of cattle pasture and occasional eucalyptus plantations. Terrain both inside and outside the park is hilly. Climate of the inland fragmented area is drier and hotter than the continuous forest, with the presence of some deciduous tree species and no tree ferns.

The sampling took advantage of a network of 1‐ha permanent forest inventory plots within the Serra do Mar State Park (established under the Biota Functional Gradients project; Joly et al. 2012) and newly established plots within fragments outside the reserve (Table 1). Data were collected from four plots within the continuous forest of the park, two in an area of intact forest (plots K and M in Joly et al. 2012), one in an area that had been selectively logged before the establishment of the park in 1977 (plot N in Joly et al. 2012), and one in a regenerating area clear‐cut for charcoal production before park establishment that is considered a mid‐stage secondary forest (Marchiori et al. 2016). These plots are referred to as intact‐K, intact‐M, logged, and secondary in the text. Two forest fragments were also sampled, one near the community of Catuçaba (fragment‐C) and one near the town of Lagoinha (fragment‐L). In fragments, two plots of 10 × 250 m were established, one at the edge (approximately 30 m from edge) and one in the interior (approx. 100 m from edge). Both fragments are adjacent to cattle pasture. While the precise history of the fragments is unknown, historical aerial imagery shows that fragment‐C has been forested since before 1962, while in fragment‐L the edge plot was pasture and the interior plot forested in 1962. All trees >4.8 cm diameter at breast height were inventoried with diameter, species identification, and co‐ordinates in the plot recorded.

**Table 1:** Details of study plots

Plot name	Plot code	Latitude/Longitude (decimal degrees)	Plot area (ha)	Fragment area (ha)	No. of profile samples	No. of hemispherical image profile samples	Dates of data collection (DD/MM, all 2015)
Intact‐K	NSV‐01	23.326 S/45.068 W	1	Continuous	12	12	26/10–05/11
Intact‐M	NSV‐02	23.328 S/45.073 W	1	Continuous	12	5	29/04–06/05
Logged	NSV‐04	23.327 S/45.076 W	1	Continuous	11	0	05/03–13/03; 28/04–29/04
Secondary	NSV‐05	23.325 S/45.094 W	1	Continuous	10	4	06/05–07/05; 20/05–21/05; 18/06–25/06
Fragment‐C	SDM‐11/SDM‐12	23.276 S/45.241 W	2 × 0.25	12.2	12	12	14/10–22/10
Fragment‐L	SDM‐17/SDM‐18	23.100 S/45.183 W	2 × 0.25	60.2	12	11	29/06–04/07

### Light profile measurements

**Figure 1:**
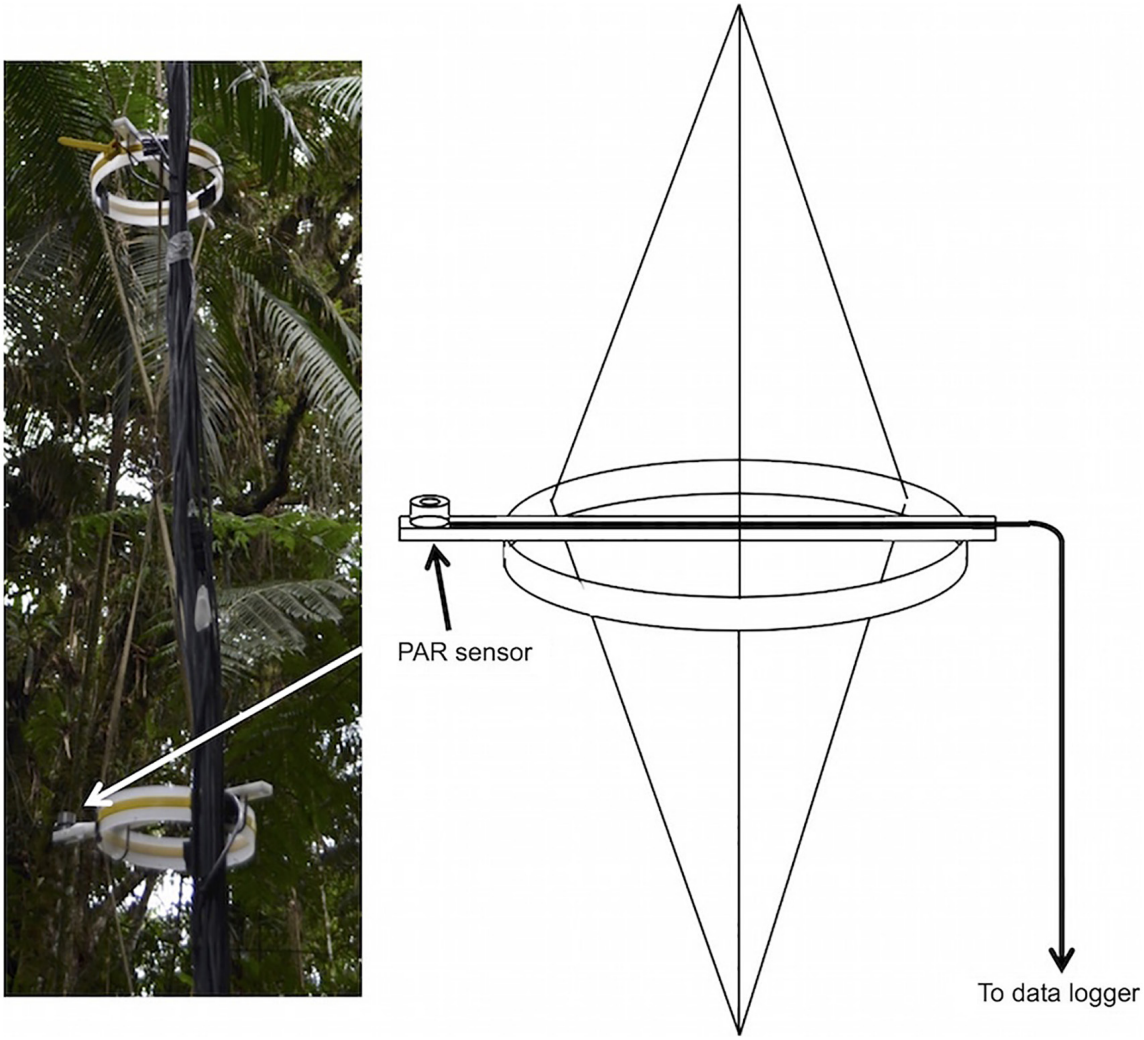
Schematic and photograph of support structure for photosynthetically active radiation (PAR) sensors. Each structure is 1 m in length.

Nineteen PAR sensors were built following the method of Fielder and Comeau (2000). For each sensor, a gallium arsenide phosphorus photodiode (G1118; Hamamatsu, Hamamatsu City, Japan) was housed in acrylic and aluminum with a cos–sine correcting diffuser. Each sensor was individually calibrated against a LI‐COR 190 quantum sensor (LI‐COR, Lincoln, Nebraska, USA). One sensor connected to a CR200 datalogger (Campbell Scientific, Logan, Utah, USA) was used as an open sky reference located either in a clearing or atop a canopy tower located in the secondary plot. All other sensors were connected to an AM16/32 multiplexer and CR800 datalogger (Campbell Scientific) to take simultaneous measurements from each sensor. Differential voltage measurements were used for the profile sensors and single‐ended measurements for the open sky sensor. To measure a profile, a thin rope was installed over a high tree branch using a Big Shot catapult (Sherrill Tree, Greensboro, North Carolina, USA) from which the sensors were suspended. Each sensor was positioned on a support structure consisting of a plastic bar bolted to a plastic ring, with each support connected to the next at 1‐m intervals with Kevlar tape (Fig. 1). Data were collected every 30 s, and the average was recorded each minute.

Fieldwork was carried out between March and October 2016. Within each plot, 10–12 locations were sampled. Each sampling point was at least 20 m away from other sampling points to ensure the independence of the light environments. For each sampling point, a 20 × 20 m subplot (10 × 10 m for fragments) was preselected under a stratified design to evenly cover each plot. Within the chosen subplot, the tallest suitable tree for measurements was selected. Trees considered suitable possessed a strong branch with a clear line of sight from the ground to enable rope and sensor installation. For one sample (point 2 in the secondary plot), the sensors were suspended from a narrow canopy tower. The profile sensor was 18 m long, so for branches higher than 18 m the sensors were pulled to the highest possible position and the bottom portion of the profile (maximum 6 m) was not measured. Each point was sampled for a minimum of one hour and a maximum of three days, always including a period of diffuse light conditions (either an overcast sky or dawn/dusk). The height of the highest leaves of the sampled tree was measured either with a laser rangefinder (Forestry Pro; Miyagi, Nikon, Japan), hypsometer (Vertex IV; Haglof, Långsele, Sweden), or visual estimation.

To produce the light profile for each point, the data were manually examined to locate time periods of measurements under diffuse conditions (in order to avoid the influence of sunflecks and sun angle on the light profiles). The mean PAR recorded by each sensor as a percentage of the open sky reference PAR (percentage of transmission, *T*) was calculated across all data points collected under diffuse conditions.

Measuring light profiles during different times of day and months of the year could lead to inaccurate determination of vertical profiles if varying sun angle influences the profile even under diffuse conditions. Strongly seasonal leaf phenology could also result in seasonal variation in the light profile. We collected light profile data continuously from November 2015 to July 2016 from a narrow canopy tower in the secondary forest plot. This showed that the light profiles determined at dusk and at dawn were very similar (Appendix S1: Fig. S1); hence, change in sun angle during the day does not affect the profile. Comparing profiles produced for different months showed that the shape of the profile was consistent over the year, but that the extent of light transmission varied over the year (Appendix S1: Fig. S2), likely due to the seasonality of leaf phenology. Light transmission was lowest, and therefore, leaf area was highest, in April, coincident with austral autumn and the transition to the drier, cooler season. The reduction in transmission between months of highest and lowest value (November–April) was low (mean 7.2% across all heights), but quite variable (standard deviation [SD] 7.6%) with higher reductions in the upper canopy (above 13 m) than lower down. Hence, the general shape of the profile does not vary over the year, but the absolute values of *T* may slightly alter.

To calculate a mean light profile for each plot, the *T* at each 1 m height above the ground was averaged across each sampling point. For fragments, data from the edge and interior transects were combined. For heights above the top of the sampled tree, *T* was assumed to be 100%. As the highest sensor was necessarily below a branch, there was an unsampled section of the light profile between the top sensor and the top of the tree. The transmission for these unsampled sections was estimated using a linear interpolation from 100% transmission at the top of the tree to *T* measured at the height of the top sensor. For profiles where the bottom sensor was above 1 m, *T* for all heights below the bottom sensor were assumed to be equal to that at the bottom sensor. In the figures, measured data points and interpolated or extrapolated data points are differentiated with different symbols.

In addition to the mean profile based on height above the ground, a mean profile using depth from the canopy top (*d*) was also produced. This means that all data points collected at the top of each sampled tree are at the same depth (0 m), rather than at different heights above the ground. We included depths up to the mean sample tree height for each plot (i.e., all data points from the canopy top downward over a vertical distance equal to the plot mean sample tree height). Where the sample tree was shorter than the plot mean sample tree height, *T* was extrapolated downward.

### Quantitative comparison of light profiles between sites

To quantify differences in the mean light profiles between sites, we estimated the extinction coefficient (*k*_z_) with height above the ground (*z*) for each mean plot profile using Eq. (1)

(1) $$T\left(z\right)=a+\exp\left(k_{z}z\right)\text{.}$$

The parameter *a* is *T* at ground level. As not all upper sections of the plot mean profile fit the exponential decay function (see *Variation in light profiles with disturbance history*), we excluded such sections from the analysis.

In order to include all the upper profile in an estimation of the extinction coefficient, we also estimate the extinction coefficient with depth (*d*) from the top of the canopy (*k*_*d*_) using Eq. (2). Note also the negative use of the extinction coefficient in comparison with Eq. (1).

(2) $$T\left(d\right)=a+\left(100-a\right)\times \exp\left(-k_{d}d\right)\text{.}$$

Parameters (*a*,* k*_*z*_, *k*_*d*_) were estimated using non‐linear least squares with the function “nls” in the statistical program R 2.15.1 (R Core Team 2012).

### Light profiles from hemispherical photographs

In addition to the data collected using PAR sensors, for 45 profiles we also collected a vertical profile of hemispherical photographs. Photographs were taken with a digital SLR camera (D3100; Nikon) and 4.5‐mm circular fisheye lens (F2.8 EX DC; Sigma, Ronkonkoma, New York, USA) using mode P and exposure compensation of −1 EV (exposure value). One of the PAR sensor support structures was adapted to serve as a cradle for the camera which was then attached to the rope; a gimbal was considered unnecessary since hand‐leveling has proven reliable for plant area index estimates from hemispherical photographs (Origo et al. 2017). The camera was programmed to take one photograph every 2 min and was pulled higher into the canopy (at *˜*2‐m intervals in the continuous forest and 1‐m intervals in the fragments) between each photograph. The 2‐min interval was typically long enough for the camera to stop rotating on the rope which was a common occurrence. The photographs were then used to estimate *T*. The images were thresholded (converted to black for vegetation and white for sky) using only the blue channel following Pfeifer et al. (2012) using the Ridler and Calvard (1978) thresholding algorithm. The thresholded images were then analyzed in the program Hemisfer (WSL, Birmensdorf, Switzerland) to determine percentage of transmission (Schleppi et al. 2007, Thimonier et al. 2010). All five annuli of the image were used, corresponding to 180° field of view. The apparatus support strings were visible in the image and were classified as vegetation. A separate analysis of eight manipulated images of strings only showed that they covered 8% of the image. As it is likely that at least some of the support strings covered vegetation area in the image, we did not attempt to correct for them. As such, the *T* estimations from the images may underestimate up to 8%, but only in cases where the transmission is very high.

All data analyses were carried out in R 2.15.1 (R Core Team 2012).

## Results and Discussion

### The shapes of light profiles

**Figure 2:**
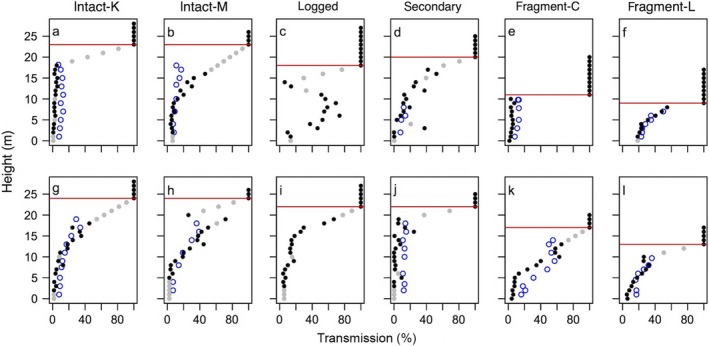
Example light profiles from each plot. Closed points, transmission measured using photosynthetically active radiation sensors; gray, interpolated or extrapolated data points; open blue points, percentage of transmission estimated from hemispherical images (no profiles from hemispherical images were available from the logged plot); red line, height of sample tree. Lower panels refer to the same plot as the upper panel.

The light profiles for each sample point are presented in Appendix S1: Fig. S3 and examples from each plot in Fig. 2. We believe this is the first study to present spatially replicated vertical light profiles along a degradation gradient. The most obvious pattern in Appendix S1: Fig. S3 is the decrease in sample tree height along the disturbance gradient, being tallest in the intact plots and shortest in the fragments (Table 2). The low canopy heights in the fragments are likely a reflection of the high level of degradation in these small fragmented forests. Other studies have shown that short‐statured, pioneer, and early successional species typically dominate Atlantic forest fragments (Tabarelli et al. 1999, Oliveira et al. 2008, Paula et al. 2011) as a result of altered seed dispersal (Costa et al. 2012) and a hotter, drier microclimate (Kapos 1989) causing biotic homogenization and a shift toward composition typical of secondary forests (Joly et al. 2014).

**Table 2:** Metrics of the light environment in plots along a degradation gradient

Plot	*T* at 1 m (mean ± SD)	*k*_*z* _± 95% CI (*R* ^2^)	*k*_*d* _± 95% CI (*R* ^2^)	Max. sample tree height (m)	Mean sample tree height (m)
Intact‐K	2.5 ± 1.7	0.190 ± 0.002 (0.98)	0.219 ± 0.012 (0.98)	28	23.1
Intact‐M	3.8 ± 2.1	0.208 ± 0.004 (0.96)	0.194 ± 0.013 (0.98)	26	21.3
Logged	6.2 ± 6.2	0.213 ± 0.010 (0.85)	0.269 ± 0.038 (0.89)	27	20.2
Secondary	2.4 ± 2.0^a^	0.246 ± 0.004 (0.98)	0.265 ± 0.007 (0.99)	25	19.2
Fragment‐C	5.8 ± 3.0	0.329 ± 0.014 (0.92)	0.290 ± 0.023 (0.98)	20	14.6
Fragment‐L	7.5 ± 5.7	0.474 ± 0.019 (0.95)	0.653 ± 0.035 (0.99)	17	10.5

Note

CI, confidence interval; SD, standard deviation.

a One value was excluded from the secondary forest mean percentage of transmission at 1 m as an outlier which had been extrapolated from a relatively high (4 m) lowest measurement (profile secondary—8).

A second point of interest is the variation in the shapes of the light profiles (Fig. 2; Appendix S1: Fig. S3). Considering all profiles, we can qualitatively split the samples into three categories: profiles that are dark throughout (Fig. 2a, e), profiles that decline from high to low light (Fig. 2b, f, k), and profiles that show inversions, or points where the available light is greater than at heights above (Fig. 2c, h, j). All three categories occur in all plots, except for dark profiles in the logged plot. The dark profiles can be considered, to a certain extent, a consequence of our sampling methodology that necessarily requires the top measurement to be below a branch. As it was not possible to sample between the top sensor to the canopy top, we miss the initial light attenuation, though we can still estimate this using our data. As we use a linear interpolation between the top of the tree and the top sensor, we could slightly overestimate *T* in the estimations at these heights, as the decline is unlikely to be completely linear, and we are assuming that there will be 100% transmission at the top of the sample tree, whereas in reality there is likely already some shading from neighboring crowns of tall trees. As these interpolated points are a minority compared with the measured points (on average 2.3 m of each profile is interpolated), this likely does not strongly influence our results. Dark profiles in the fragments were typically found in subplots with a dense liana layer covering the tree crowns. Lianas are known to be particularly abundant in disturbed areas (Schnitzer and Bongers 2011), and high abundances of small lianas have been found in other studies of forest fragments (Oliveira‐Filho et al. 1997, Laurance et al. 2001, Farah et al. 2014). This high liana abundance can have a strong impact on the light environment, restricting the penetration of light even very close to the canopy top, supporting other work that showing that lianas can reduce forest productivity (van der Heijden et al. 2015, Lai et al. 2017). While lianas, or high epiphyte loads which are common in the continuous forest, may also be a cause of the dark profiles in the continuous plots, our inability to reach the canopy top was more prevalent in the continuous forest than in the fragments, and the influence of the interpolations is likely higher. Despite this limitation, the dark profiles are still of interest as they show that in some cases *T* is already very low just below the canopy top; light transmission can be less than 5% as high up as 18 m, or 5 m below the top of the crown (Fig. 2a). This is due to the dense upper canopy absorbing substantial light—up to 95% in this study.

The profiles with inversions were somewhat unexpected as they do not conform to the broadly assumed exponentially decaying light availability profile. Some of the inverted profiles may be a result of the methodology with direct shading of the top sensor by the branch from which the sensors were suspended (Fig. 2h). However, in others the inversion occurs further down (Fig. 2c). While there are little data available on light profiles with spatially extensive sampling from other sites, inversions in the light profile have been observed in intact tropical forest in Venezuela (Anhuf and Rollenbeck 2001) and in temperate coniferous (Parker 1997) and deciduous (Parker et al. 1996) forests. These inversions are due to incoming light through lateral canopy gaps. In this study, the inversions are a particularly common occurrence in the logged plot; selective logging removes large crowns creating gaps in the upper canopy.

### Variation in light profiles with disturbance history

**Figure 3:**
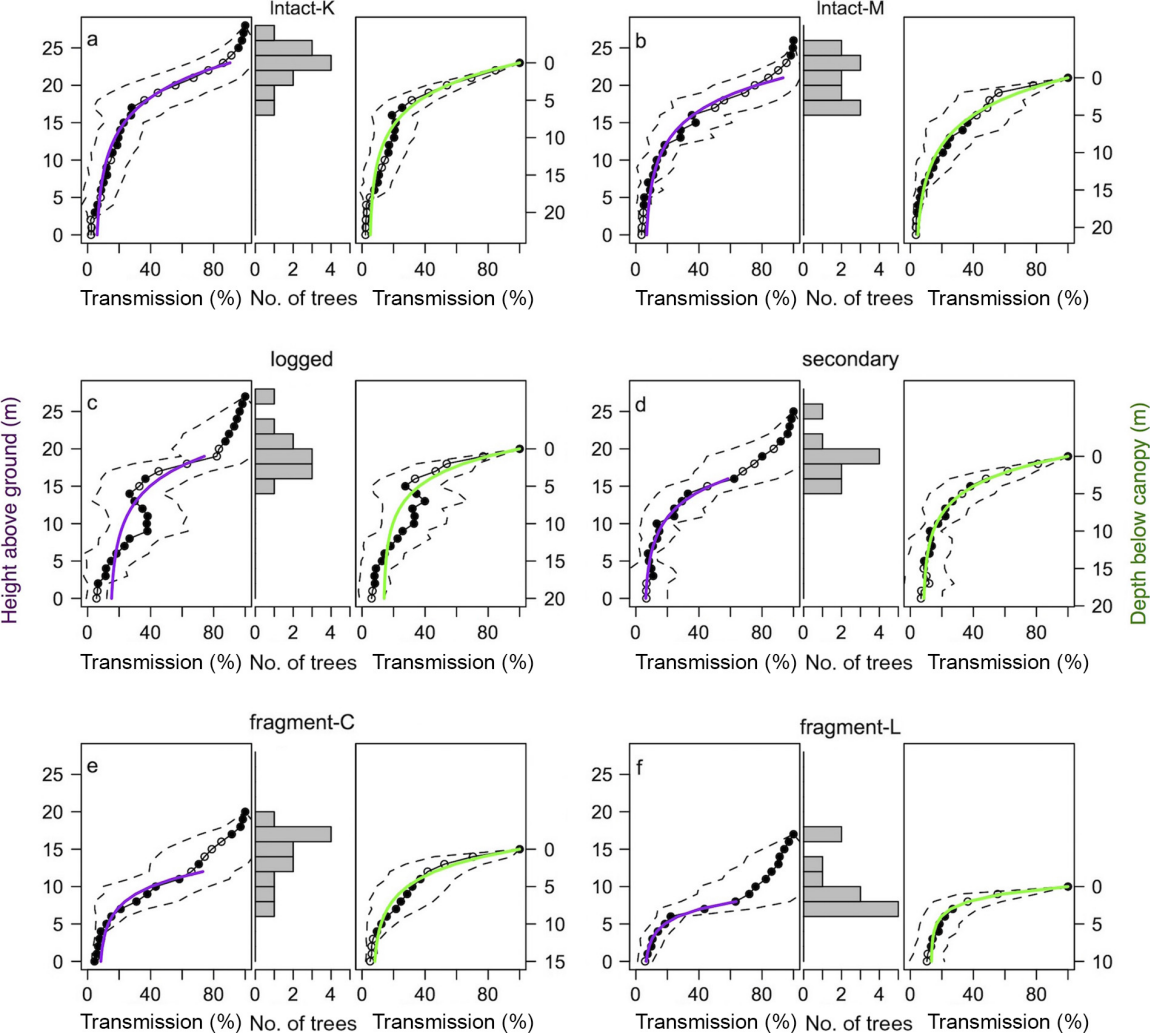
Observed mean light profiles with height (left panel) and depth below the canopy (right panel) for forest plots along a disturbance gradient. Open circles represent data points where three or more subplot profile measurements are based on interpolations or extrapolations due to the estimation of the top or bottom sections of the profiles. Dashed lines show standard deviation between observed transmission values of different samples within the plot. Purple line, extinction coefficient fit to mean light profile using height above the ground. Green line, extinction coefficient fit to mean light profile using depth from top of canopy. Middle panels show histograms of the sample tree heights within each plot.

A feature of the mean height–*T* profile produced for each plot (Fig. 3) is the initial small declines in available light before rapid light attenuation occurs. This is due to the averaging across individual profiles with different canopy heights, and the extent of this effect reflects spatial heterogeneity in tree height. In contrast, the mean *T* profiles are not affected by heterogeneity in tree height and show exponential decline in light from canopy top downward. In plots where there are few tall sample trees and many shorter ones (e.g., logged and fragment‐L), this slow attenuation in height‐based profiles continues further down the canopy. Interestingly, fragment‐C also shows this slow decline, but without the characteristic pattern of few large trees to explain it. The distribution of sample tree size is quite different between fragment‐C and fragment‐L, yet fragment‐C does not have higher attenuation in its upper canopy. This may be due to an effect of season of sampling as, while in the continuous plots all species are evergreen, deciduous species are present in the fragments and some had lost their leaves at the time of sampling fragment‐C (end of the dry season). Leaf shedding alters the patterns of light below tree crowns (Gandolfi et al. 2007) and hence likely influenced our measurements in fragment‐C. In fragment‐L, there is strong light attenuation between 6 and 8 m height, just below the median sample tree height. In this plot, especially in the edge transect, trees were similarly sized with a homogenous canopy layer around 8 m high, resulting in strong light absorption at this height.

The mean profiles of both height and depth show that the inversions seen in the individual profiles are maintained in mean profile for the logged plot (Fig. 3c). While inversions are to be expected in any forest with a heterogeneous canopy, the pattern should not be visible in the mean profile given sufficient sampling; otherwise, it would suggest a source of light within the forest (Parker 1997). The fact that this has occurred in the logged forest only suggests that the canopy in this plot is more heterogeneous than all other forest types studied. This prevalence of inverted profiles in the logged plots is despite the fact that the logging occurred over 40 yr ago. One might expect that gaps produced by logging (or natural tree death) would be filled in over this timescale by the remaining trees growing laterally (Young and Hubbell 1991) or new trees filling the space, thereby removing these light gaps. However, recovery of forest structure (biomass and/or basal area) in selectively logged forests takes considerable time, with estimations in the order of decades, ranging from 10 to ~100 yr depending on the logging intensity (Blanc et al. 2009, Huang and Asner 2010, Bonnell et al. 2011, West et al. 2014, Rutishauser et al. 2015). In this case, the biomass of the logged plot (274 Mg/ha; Vieira et al. 2011) is similar to three intact plots (including the two in this study) at the site (242–323 Mg/ha; Vieira et al. 2011), suggesting that the biomass at least has recovered in this plot (though the pre‐logging biomass of the plot is unknown). Rutishauser et al. (2016) show that the diameter–height allometry of remnant trees in logged forest varies from intact forest, with shorter trees for a given diameter in logged forest, an effect that was present even 25 yr after the disturbance. They suggest that the height reduction is a consequence of crown development at lower heights due to the altered light environment. This reduction in height growth could maintain canopy heterogeneity rather than filling in gaps.

Both of the intact plots show a similar pattern, with a sharp initial decline in light through the upper canopy and a slower decline below (Fig. 3a, b). The profile shapes of the intact plots are similar to those directly measured from other sites in Venezuela (Anhuf and Rollenbeck 2001) and estimated from LiDAR in the central Amazon (Stark et al. 2012).

Interestingly, within the continuous forest, the shape of the secondary forest profile is more similar to the intact forest than the logged forest (Fig. 3a–d). Below 10 m height, there is very little difference between the secondary and intact profiles while there is considerably more light transmission in the logged plot, with significantly higher *T* at 10 m height in logged plot 37.6% ± 23.1% (mean ± SD) than in the secondary, intact‐K, and intact‐M plots with 13.3 ± 9.9, 14.8 ± 12.3, and 13.3 ± 9.1, respectively (ANOVA, *F *= 7.1, df = 3, *P *<* *0.001 with logged significantly to other plots in Tukey's post hoc test). This is surprising considering that regrowth from clear felling could be considered a greater disturbance than selective logging and that the secondary plot contains ~68% of the intact plot biomass (Marchiori et al. 2016). This shows that despite recovery of some characteristics (e.g., biomass), logged forest can still show structural differences long after the logging event. Further, despite the difference in biomass between the secondary and intact plots, the conditions for the understory may be quite similar. While logged forests will have a composition more similar to intact forest than secondary forest (Gibson et al. 2011), the mid‐canopy light conditions can be brighter and may be less conducive to the growth of shade‐tolerant species than the darker mid‐canopy of a recovering secondary forest. Further understanding is needed on patterns of structural forest recovery after disturbance and the consequences for the vertical light environment and tree growth.

To quantitatively compare light profiles between plots, we estimated the extinction coefficient (*k*_*z*_) of light attenuation with canopy height (using only the profile data at and below rapid light attenuation) and with canopy depth (*k*_*d*_, using only the profile data from the canopy top to the mean tree height; Table 2, Fig. 3). The height‐based extinction coefficient increased along the disturbance gradient, intact‐K < intact‐M < logged < secondary < fragment‐C < fragment‐L, and from examination of the 95% confidence interval around *k*_*z*_ estimates, the extinction coefficients varied significantly between all plots except intact‐M and logged. The variance explained (*R* ^2^) by *k*_*z*_ for the logged plot was low compared to the other plots due to the inversion section of the logged profile. Results based on *k*_*d*_ were similar to *k*_*z*_, but without significant differences in *k*_*d*_ between logged, secondary, and fragment‐C. Both *k*_*z*_ and *k*_*d*_ were significantly negatively correlated with canopy height (Pearson's correlation, *k*_*z*_ − *r *= −0.98, *P *<* *0.001, *k*_*d*_ − *r *= −0.88, *P *=* *0.02; Table 2). This reflects the fact that *T* of the understory is low at all sites, but the canopy heights are quite different; at the lower canopy forests, a similar total amount of light is absorbed to tall forests, but over a shorter vertical distance, and hence, *k*_*z*_ and *k*_*d*_ are higher. This may indicate that many small dense crowns can absorb a similar amount of light to fewer large but sparse canopies. *K*_*d*_ is probably a better descriptor of differences in light extinction between sites than *k*_*z*_ because it includes all data points from the top of the canopy.

To compare with data typically reported in other studies, we present *T* at 1 m above the ground in each plot (Table 2). The intact and secondary plots show low transmission, with higher transmission in the logged and fragment plots. The differences between plots are significant (ANOVA, *F *=* *3.2, df = 5, *P *=* *0.013); however, only intact‐K and fragment‐L were significantly different in a post hoc test (Tukey, *P *=* *0.035). The lack of significant differences is partly a result of high variance within plots, but does highlight that light measurements of the understory cannot necessarily inform about the light environment above that sample point. For example, even with a similar leaf area index below the canopy, the light profiles of two Amazonian forests showed different patterns (Stark et al. 2012).

### Comparison of profiles from PAR sensors and hemispherical images

**Figure 4:**
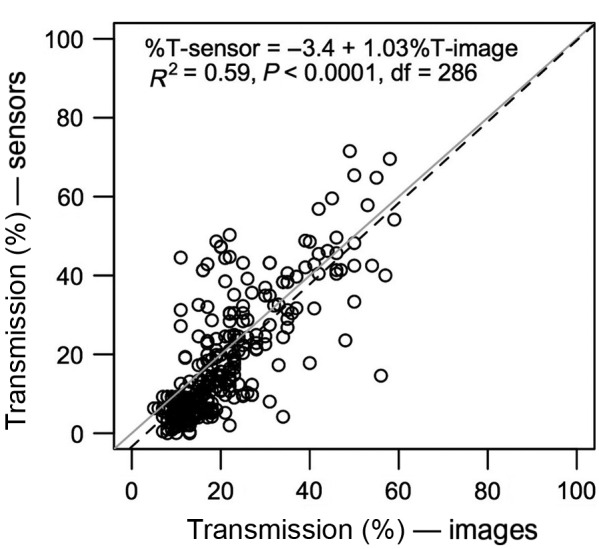
Relationship between transmission measured from photosynthetically active radiation sensors and transmission estimated from hemispherical images. Gray line, *y *= *x*; black dashed line, regression line.

Transmission as estimated from the PAR sensors and the vertical profiles of hemispherical photographs show good agreement (Figs. 2, 4; Appendix S1: Fig. S4). The *R* ^2^ of the relationship between *T* measured with the PAR sensors and *T* estimated from the hemispherical images was 0.59 (Fig. 4). Of the 44 individual samples that had both sensor and image profiles, the sensor and image transmission values were significantly correlated in 25 (Appendix S1: Fig. S4). Those that were not significantly correlated typically had few images and/or showed little within profile variation. Even for those that were not correlated, the transmission values were similar.

We believe this is the first study to estimate forest vertical light environments using profiles of hemispherical images. Accessing the forest canopy is prohibitively difficult, and hence, there are few data on vertical patterns of canopy structure and light (Parker 1995). Previous attempts (in temperate and boreal regions) have typically used cameras mounted on telescoping poles that can only reach limited heights (e.g., 16 m, Wang et al. 1992; 10 m, Zhu et al. 2003; 6.8 m, Domke et al. 2007), or from a single sample point using a tower (Strachan and McCaughey 1996). Recent work has shown such knowledge can aid understanding of tropical forest dynamics (Stark et al. 2012, 2015), and light interception is a key aspect of vegetation models, from individual‐based forest simulators to dynamic global vegetation models. While the burgeoning field of forest canopy science (Nadkarni et al. 2011) will no doubt continue to be influenced by the high‐resolution data available from LiDAR, such technology is still expensive and intensive data processing and analysis is required to determine light environments from it. The method we developed during this study enables rapid estimation of vertical light profiles from the ground that can be repeated within and between sites to encompass spatial heterogeneity. The method is inexpensive—the camera and lens used here costing around £1000 (less than the data loggers required for the PAR sensors)—and the equipment is readily available. This could be a very useful method to extend our understanding of vertical profiles of light (or leaf area) from more locations, either in its own right or to compliment LiDAR‐based data acquisition.

## Conclusions

This study has measured patterns of vertical light penetration along a disturbance gradient in the biodiversity hot spot of the Atlantic forest. Our results show that there is spatial variation in vertical light patterns both within (Fig. 2) and between plots with different disturbance histories (Fig. 3). Logging in particular has an impact on vertical light patterns, with greater within plot heterogeneity and inversions in the profile due to lateral light from upper canopy gaps. This pattern is still present despite 40 yr of recovery from the logging event. We show that despite similarities in transmission to the forest floor across all disturbances there are differences in vertical structure and light extinction (Table 2) that may influence the light availability, and hence growth, of mid‐canopy trees. We also show the importance of non‐tree life forms (e.g., lianas) for light transmission in forest fragments. Finally, we recommend the rapid and inexpensive methodology using hemispherical photographs for the characterization of forest vertical light regimes, which are rarely measured.

## Data Availability

The data are publicly available through deposit to NERC's Environmental Information Data Centre (Fauset et al. 2017).

## References

[ecs22002-bib-0001] Alton, P. B. , P. R. North , and S. O. Los . 2007 The impacts of diffuse sunlight on canopy light‐use efficiency, gross photosynthetic product and net ecosystem exchange in three forest biomes. Global Change Biology 13:776–787.

[ecs22002-bib-0002] Anhuf, D. , and R. Rollenbeck . 2001 Canopy structure of the Rio Surumoni rain forest (Venezuela) and its influence on microclimate. Ecotropica 7:21–32.

[ecs22002-bib-0003] Berenguer, E. , J. Ferreira , T. A. Gardner , L. E. O. C. Aragão , O. C. De Carmargo , C. E. Cerri , M. Durigan , R. C. De Oliveira Junior , I. C. G. Vieira , and J. Barlow . 2014 A large‐scale field assessment of carbon stocks in human‐modified tropical forests. Global Change Biology 20:3713–3726. 24865818 10.1111/gcb.12627

[ecs22002-bib-0004] Binkley, D. , O. C. Campoe , M. Gspaltl , and D. I. Forrester . 2013 Light absorption and use efficiency in forests: why patterns differ for trees and stands. Forest Ecology and Management 288:5–13. 10.1016/j.foreco.2011.11.044 PMC4268600 25540477

[ecs22002-bib-0005] Blanc, L. , M. Echard , B. Herault , D. Bonal , E. Marcon , J. Chave , and C. Baraloto . 2009 Dynamics of aboveground carbon stocks in a selectively logged tropical forest. Ecological Applications 19:1397–1404. 19769089 10.1890/08-1572.1

[ecs22002-bib-0006] Bonnell, T. R. , R. Reyna‐Hurtado , and C. A. Chapman . 2011 Post‐logging recovery time is longer than expected in an East African tropical forest. Forest Ecology and Management 261:855–864.

[ecs22002-bib-0007] Canham, C. D. , J. S. Denslow , W. J. Platt , J. R. Runkle , T. A. Spies , and P. S. White . 1990 Light regimes beneath closed canopies and tree‐fall gaps in temperate and tropical forests. Canadian Journal of Forest Research 20:620–631.

[ecs22002-bib-0008] Chazdon, R. L. , and N. Fetcher . 1984 Photosynthetic light environments in a lowland tropical rain forest in Costa Rica. Journal of Ecology 72:553–564.

[ecs22002-bib-0009] Chen, A. , J. W. Lichstein , J. L. D. Osnas , and S. W. Pacala . 2014 Species‐independent down‐regulation of leaf photosynthesis and respiration in response to shading: evidence from six temperate tree species. PLoS ONE 9:e91798. 24727745 10.1371/journal.pone.0091798 PMC3984078

[ecs22002-bib-0010] Clark, D. B. , P. C. Olivas , S. F. Oberbauer , D. A. Clark , and M. G. Ryan . 2008 First direct landscape‐scale measurement of tropical rain forest leaf area index, a key driver of global primary productivity. Ecology Letters 11:163–172. 18031553 10.1111/j.1461-0248.2007.01134.x

[ecs22002-bib-0011] Costa, J. B. P. , F. L. P. Melo , B. A. Santos , and M. Tabarelli . 2012 Reduced availability of large seeds constrains Atlantic forest regeneration. Acta Oecologica 39:61–66.

[ecs22002-bib-0012] Denslow, J. S. , and S. G. Guzman . 2000 Variation in stand structure, light and seedling abundance across a tropical moist forest chronosequence, Panama. Journal of Vegetation Science 11:201–212.

[ecs22002-bib-0013] Didham, R. K. , and J. H. Lawton . 1999 Edge structure determines the magnitude of changes in microclimate and vegetation structure in tropical forest fragments. Biotropica 31:17–30.

[ecs22002-bib-0014] Dirzo, R. , and P. H. Raven . 2003 Global state of biodiversity and loss. Annual Review of Environment and Resources 28:137–167.

[ecs22002-bib-0015] Domke, G. M. , J. P. Caspersen , and T. A. Jones . 2007 Light attenuation following selection harvesting in northern hardwood forests. Forest Ecology and Management 239:182–190.

[ecs22002-bib-0016] Ewers, R. M. , and C. Banks‐Leite . 2013 Fragmentation impairs the microclimate buffering effect of tropical forests. PLoS ONE 8:e58093. 23483976 10.1371/journal.pone.0058093 PMC3587424

[ecs22002-bib-0017] Farah, F. T. , R. R. Rodrigues , F. A. M. Santos , J. Y. Tamashiro , G. J. Shepherd , T. Siqueira , J. L. F. Batista , and B. J. F. Manly . 2014 Forest destructuring as revealed by the temporal dynamics of fundamental species: case study of Santa Genebra forest in Brazil. Ecological Indicators 37:40–44.

[ecs22002-bib-0018] Fauset, S. , M. U. Gloor , M. P. M. Aidar , H. C. Freitas , N. M. Fyllas , C. A. Joly , M. A. Marabesi , A. L. C. Rochelle , A. Shenkin , and S. A. Vieira . 2017 Vertical profile data of light transmission in Atlantic forests along a disturbance gradient. NERC Environmental Information Data Centre. https://doi.org/10.5285/4f3cf9f6-d7e5-4ae0-87c9-064b4e66a92a

[ecs22002-bib-0019] Fielder, P. , and P. Comeau . 2000 Construction and testing of an inexpensive PAR sensor. Working Paper 53/2000. Research Branch, Ministry of Forests, Victoria, British Columbia, Canada.

[ecs22002-bib-0020] Gandolfi, S. , C. A. Joly , and R. R. Rodrigues . 2007 Permeability–impermeability: canopy trees as biodiversity filters. Scientia Agricola 64:433–438.

[ecs22002-bib-0021] Gibson, L. , et al. 2011 Primary forests are irreplaceable for sustaining tropical biodiversity. Nature 478:378–381. 21918513 10.1038/nature10425

[ecs22002-bib-0022] Huang, M. , and G. P. Asner . 2010 Long‐term carbon loss and recovery following selective logging in Amazon forests. Global Biogeochemical Cycles 24:GB3028.

[ecs22002-bib-0023] Joly, C. A. , J. P. Metzger , and M. Tabarelli . 2014 Experiences from the Brazilian Atlantic Forest: ecological findings and conservation initiatives. New Phytologist 204:459–473. 25209030 10.1111/nph.12989

[ecs22002-bib-0024] Joly, C. A. , et al. 2012 Florística e fitossociologia em parcelas permanents da Mata Atlântica do sudeste do Brasil ao longo de um gradient altitudinal. Biota Neotropica 12:123–145.

[ecs22002-bib-0025] Kapos, V. 1989 Effects of isolation on the water status of forest patches in the Brazilian Amazon. Journal of Tropical Ecology 5:173–185.

[ecs22002-bib-0026] Kitajima, K. , S. S. Mulkey , and S. J. Wright . 2005 Variation in crown light utilization characteristics among tropical canopy trees. Annals of Botany 95:535–547. 15585541 10.1093/aob/mci051 PMC4246798

[ecs22002-bib-0027] Lai, H. R. , J. S. Hall , B. L. Turner , and M. van Breugel . 2017 Liana effects on biomass dynamics strengthen during secondary forest succession. Ecology 98:1062–1070. 28072458 10.1002/ecy.1734

[ecs22002-bib-0028] Laurance, W. F. , S. G. Laurance , L. V. Ferreira , J. M. Rankin‐de Merona , C. Gascon , and T. E. Lovejoy . 1997 Biomass collapse in Amazonian forest fragments. Science 278:1117–1118.

[ecs22002-bib-0029] Laurance, W. F. , D. Pérex‐Salicrup , P. Delamônica , P. M. Fearnside , S. D'Angelo , A. Jerozolinski , L. Pohl , and T. E. Lovejoy . 2001 Rain forest fragmentation and the structure of Amazonian liana communities. Ecology 82:105–116.

[ecs22002-bib-0030] Laurance, W. F. , et al. 2011 The fate of Amazonian forest fragments: a 32‐year investigation. Biological Conservation 144:56–67.

[ecs22002-bib-0031] Lebrija‐Trejos, E. , E. A. Pérez‐García , J. A. Meave , L. Poorter , and F. Bongers . 2011 Environmental changes during secondary succession in a tropical dry forest in Mexico. Journal of Tropical Ecology 27:477–489.

[ecs22002-bib-0032] Lefsky, M. A. , W. B. Cohen , G. G. Parker , and D. J. Harding . 2002 Lidar remote sensing for ecosystem studies. BioScience 52:19–30.

[ecs22002-bib-0033] Maass, J. M. , J. M. Vose , W. T. Swank , and A. Martínez‐Yrízar . 1995 Seasonal changes of leaf area index (LAI) in a tropical deciduous forest in west Mexico. Forest Ecology and Management 74:171–180.

[ecs22002-bib-0034] Magnago, L. F. S. , M. F. Rocha , L. Meyer , S. V. Martins , and J. A. A. Meira‐Neto . 2015 Microclimatic conditions at forest edges have significant impacts on vegetation structure in large Atlantic forest fragments. Biodiversity and Conservation 24:2305–2318.

[ecs22002-bib-0035] Marchiori, N. M. , H. R. Rocha , J. Y. Tamashiro , and M. P. M. Aidar . 2016 Tree community composition and aboveground biomass in a secondary Atlantic forest, Serra do Mar state park, São Paulo, Brazil. CERNE 22:501–514.

[ecs22002-bib-0036] Michalski, F. , I. Nishi , and C. A. Peres . 2007 Distance‐mediated drift in tree functional groups in Amazonian forest fragments. Biotropica 39:691–701.

[ecs22002-bib-0037] Montgomery, R. A. , and R. L. Chazdon . 2001 Forest structure, canopy architecture, and light transmittance in tropical wet forests. Ecology 82:2707–2718.

[ecs22002-bib-0038] Nadkarni, N. M. , G. G. Parker , and M. D. Lowman . 2011 Forest canopy studies as an emerging field of science. Annals of Forest Science 68:217–224.

[ecs22002-bib-0039] Newmark, W. D. 2001 Tanzanian forest edge microclimatic gradients: dynamic patterns. Biotropica 33:2–11.

[ecs22002-bib-0040] Nicotra, A. B. , R. L. Chazdon , and S. V. B. Iriarte . 1999 Spatial heterogeneity of light and woody seedling regeneration in tropical wet forests. Ecology 80:1908–1926.

[ecs22002-bib-0041] Oliveira, M. A. , A. M. M. Santos , and M. Tabarelli . 2008 Profound impoverishment of the large‐tree stand in a hyper‐fragmented landscape of the Atlantic forest. Forest Ecology and Management 256:1910–1917.

[ecs22002-bib-0042] Oliveira‐Filho, A. T. , and M. A. L. Fontes . 2000 Patterns of floristic differentiation among Atlantic Forests in southeastern Brazil and the influence of climate. Biotropica 32:793–810.

[ecs22002-bib-0043] Oliveira‐Filho, A. T. , J. M. Mello , and J. R. S. Scolforo . 1997 Effects of past disturbance and edges on tree community structure and dynamics within a fragment of tropical semideciduous forest in south‐eastern Brazil over a five‐year period (1987–1992). Plant Ecology 131:45–66.

[ecs22002-bib-0044] Origo, N. , K. Calders , J. Nightingale , and M. Disney . 2017 Influence of leveling technique on the retrieval of canopy structural parameters from digital hemispherical photography. Agricultural and Forest Meteorology 237:143–249.

[ecs22002-bib-0045] Osazuwa‐Peters, O. L. , C. A. Chapman , and A. E. Zanne . 2015 Selective logging: Does the imprint remain on tree structure and composition after 45 years? Conservation Physiology 3 https://doi.org/10.1093/conphys/cov012 10.1093/conphys/cov012 PMC4778436 27293697

[ecs22002-bib-0046] Pan, Y. , et al. 2011 A large and persistent carbon sink the world's forests. Science 333:988–993. 21764754 10.1126/science.1201609

[ecs22002-bib-0047] Parker, G. G. 1995 Structure and microclimate of forest canopies Pages 73–106 *in* Lowman M. D. and Nadkarni N. M., editors. Forest canopies. Academic Press, San Diego, California, USA.

[ecs22002-bib-0048] Parker, G. G. 1997 Canopy structure and light environment of an old growth Douglas‐fir/western hemlock forest. Northwest Science 71:261–270.

[ecs22002-bib-0049] Parker, G. G. , M. M. Davis , and S. M. Chapotin . 2002 Canopy light transmittance in Douglas‐fir‐western hemlock stands. Tree Physiology 22:147–157. 11830411 10.1093/treephys/22.2-3.147

[ecs22002-bib-0050] Parker, G. G. , M. A. Lefsky , and D. J. Harding . 2001 Light transmittance in forest canopies determined using airborne laser altimetry and in‐canopy quantum measurements. Remote Sensing of Environment 76:298–309.

[ecs22002-bib-0051] Parker, G. G. , P. J. Stone , and D. Bowers . 1996 A balloon for microclimate observations within the forest canopy. Journal of Applied Ecology 33:173–177.

[ecs22002-bib-0052] Parker, G. G. , C. Tinoco‐Ojanguren , A. Martínez‐Yrízar , and M. Maass . 2005 Seasonal balance and vertical pattern of photosynthetically active radiation within canopies of a tropical dry deciduous forest ecosystem in Mexico. Journal of Tropical Ecology 21:283–295.

[ecs22002-bib-0053] Paula, M. D. , C. P. A. Costa , and M. Tabarelli . 2011 Carbon storage in a fragmented landscape of Atlantic forest: the role played by edge‐affected habitats and emergent trees. Tropical Conservation Science 3:349–358.

[ecs22002-bib-0054] Pfeifer, M. , A. Gonsamo , M. Disney , P. Pellikka , and R. Marchant . 2012 Leaf area index for biomes of the Eastern Arc Mountains: Landsat and SPOT observations along precipitation and altitude gradients. Remote Sensing of Environment 118:103–115.

[ecs22002-bib-0055] Poorter, L. , L. Bongers , and F. Bongers . 2006 Architecture of 54 moist‐forest tree species: traits, trade‐offs, and functional groups. Ecology 87:1289–1301. 16761607 10.1890/0012-9658(2006)87[1289:aomtst]2.0.co;2

[ecs22002-bib-0056] R Development Core Team . 2012 R: a language and environment for statistical computing. R Foundation for Statistical Computing, Vienna, Austria.

[ecs22002-bib-0057] Ribeiro, M. C. , J. P. Metzger , A. C. Martensen , F. L. Ponzoni , and M. M. Hirota . 2009 The Brazilian Atlantic Forest: How much is left, and how is the remaining forest distributed? Implications for conservation. Biological Conservation 142:1141–1153.

[ecs22002-bib-0058] Rich, P. M. , D. B. Clark , D. A. Clark , and S. F. Oberbauer . 1993 Long‐term study of solar radiation regimes in a tropical wet forest using quantum sensors and hemispherical photography. Agricultural and Forest Meteorology 65:107–127.

[ecs22002-bib-0059] Ridler, T. W. , and S. Calvard . 1978 Picture thresholding using an iterative selection method. IEEE Transactions on Systems, Man, and Cybernetics SMC‐8:630–632.

[ecs22002-bib-0060] Rosado, B. H. P. , R. S. Oliveira , and M. P. M. Aidar . 2010 Is leaf water repellency related to vapor pressure deficit and crown exposure in tropical forests? Acta Oecologica 36:645–649.

[ecs22002-bib-0061] Rutishauser, E. , B. Hérault , P. Petronelli , and P. Sist . 2016 Tree height reduction after selective logging in a tropical forest. Biotropica 48:285–289.

[ecs22002-bib-0062] Rutishauser, E. , et al. 2015 Rapid tree carbon stock recovery in managed Amazonian forests. Current Biology 25:R787. 26394096 10.1016/j.cub.2015.07.034

[ecs22002-bib-0063] Schleppi, P. , M. Conedera , I. Sedivy , and A. Thimonier . 2007 Correcting non‐linearity and slope effects in the estimation of the leaf area index of forests from hemispherical photographs. Agricultural and Forest Meteorology 144:236–242.

[ecs22002-bib-0064] Schnitzer, S. A. , and F. Bongers . 2011 Increasing liana abundance and biomass in tropical forests: emerging patterns and putative mechanisms. Ecology Letters 14:397–406. 21314879 10.1111/j.1461-0248.2011.01590.x

[ecs22002-bib-0065] Stark, S. C. , B. J. Enquist , S. R. Saleska , V. Leitold , J. Schietti , M. Longo , L. F. Alves , P. B. Carmago , and R. C. Oliveira . 2015 Linking canopy leaf area and light environments with tree size distributions to explain Amazon forest demography. Ecology Letters 18:636–645. 25963522 10.1111/ele.12440

[ecs22002-bib-0066] Stark, S. C. , et al. 2012 Amazon forest carbon dynamics predicted by profiles of canopy leaf area and light environment. Ecology Letters 15:1406–1414. 22994288 10.1111/j.1461-0248.2012.01864.x

[ecs22002-bib-0067] Strachan, I. B. , and J. H. McCaughey . 1996 Spatial and vertical leaf area index of a deciduous forest resolved using the LAI‐2000 plant canopy analyzer. Forest Science 42:176–181.

[ecs22002-bib-0068] Tabarelli, M. , W. Mantovani , and C. A. Peres . 1999 Effects of habitat fragmentation on plant guild structure in the montane Atlantic forest of southeastern Brazil. Biological Conservation 91:119–127.

[ecs22002-bib-0069] Thimonier, A. , I. Sedivy , and P. Schleppi . 2010 Estimating leaf area index in different types of mature forest stands in Switzerland: a comparison of methods. European Journal of Forest Research 129:543–562.

[ecs22002-bib-0070] Torquebiau, E. F. 1988 Photosynthetically active radiation environment, patch dynamics and architecture in a tropical rainforest in Sumatra. Australian Journal of Plant Physiology 15:327–342.

[ecs22002-bib-0071] van der Heijden, G. , J. S. Powers , and S. A. Schnitzer . 2015 Lianas reduce carbon accumulation and storage in tropical forests. Proceedings of the National Academy of Sciences USA 112:13267–13271. 10.1073/pnas.1504869112 PMC4629347 26460031

[ecs22002-bib-0072] Veloso, H. P. , A. L. R. Rangel Filho , and J. C. A. Lima . 1991 Classificação da vegetação brasileira, adaptada a um sistema universal. Instituto Brasileiro de Geografia e Estatítica, Rio de Janeiro, Brazil.

[ecs22002-bib-0073] Vieira, S. A. , et al. 2011 Stocks of carbon and nitrogen and partitioning between above‐ and belowground pools in the Brazilian coastal Atlantic Forest elevation range. Ecology and Evolution 1:421–434. 22393511 10.1002/ece3.41 PMC3287305

[ecs22002-bib-0074] Wang, Y. S. , D. R. Miller , J. M. Welles , and G. M. Heisler . 1992 Spatial variability of canopy foliage in an oak forest estimated with fisheye sensors. Forest Science 38:854–865.

[ecs22002-bib-0075] West, T. A. P. , E. Vidal , and F. E. Putz . 2014 Forest biomass recovery after conventional and reduced‐impact logging in Amazonian Brazil. Forest Ecology and Management 314:59–63.

[ecs22002-bib-0076] Wirth, R. , B. Weber , and R. J. Ryel . 2001 Spatial and temporal variability of canopy structure in a tropical moist forest. Acta Oecologica 22:1–10.

[ecs22002-bib-0077] Yamada, T. , A. Yoshioka , M. Hashim , N. Liang , and T. Okuda . 2014 Spatial and temporal variations in the light environment in a primary and selectively logged forest long after logging in Peninsular Malaysia. Trees 28:1355–1365.

[ecs22002-bib-0078] Yoda, K. 1974 Three‐dimensional distribution of light intensity in a tropical rain forest of west Malaysia. Japanese Journal of Ecology 24:247–254.

[ecs22002-bib-0079] Young, T. P. , and S. P. Hubbell . 1991 Crown asymmetry, treefalls, and repeat disturbance of broad‐leaved forest gaps. Ecology 72:1464–1471.

[ecs22002-bib-0080] Zhu, J. J. , T. Matsuzaki , and Y. Gonda . 2003 Optical stratification porosity as a measure of vertical canopy structure in a Japanese coastal forest. Forest Ecology and Management 173:89–104.

