# Supplementary material for: Tropical forest light regimes in a human‐modified landscape
Source: Ecosphere. 2017 Nov 22;8(11):e02002. doi: 10.1002/ecs2.2002 (PMC5731677; doi:10.1002/ecs2.2002)
Supplement: Supplementary file 1 [file ECS2-8-na-s001.pdf]

Appendix S1 – Supplementary Figures

These figures are associated with the article 'Tropical forest light regimes in a human-modified landscape', Fauset et al., Ecosphere.

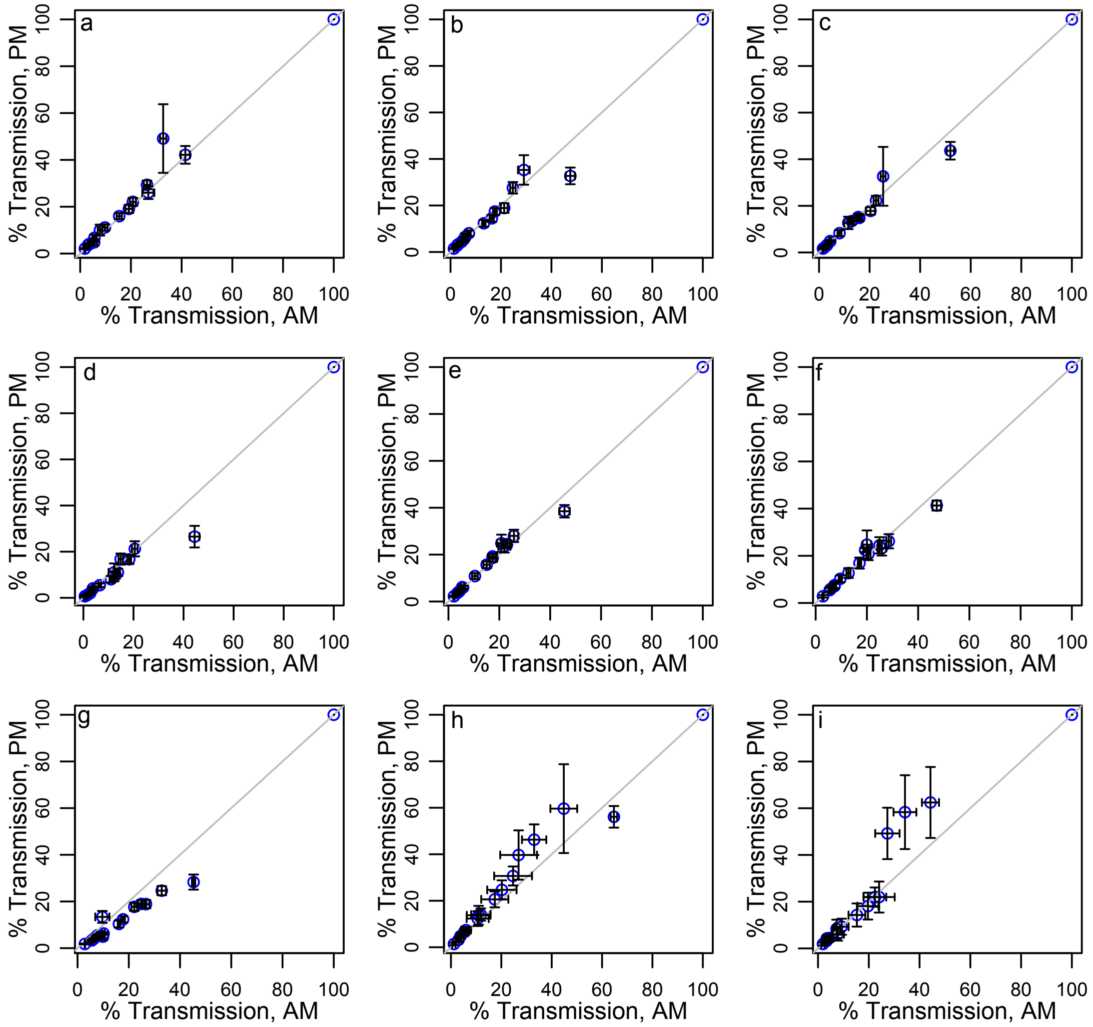

Figure S1. % transmission estimated at dawn (AM) and dusk (PM) from a single light profile collected in a) January 2016 over 31 days, b) February 2016 over 29 days, c) March 2016 over 30 days, d) April 2016 over 30 days, e) May 2016 over 31 days, f) June 2016 over 30 days, g) July 2016 over 15 days, h) November 2015 over 15 days, i) December 2015 over 9 days. Error bars show 95 % confidence interval across sampling days. Grey line = 1:1.

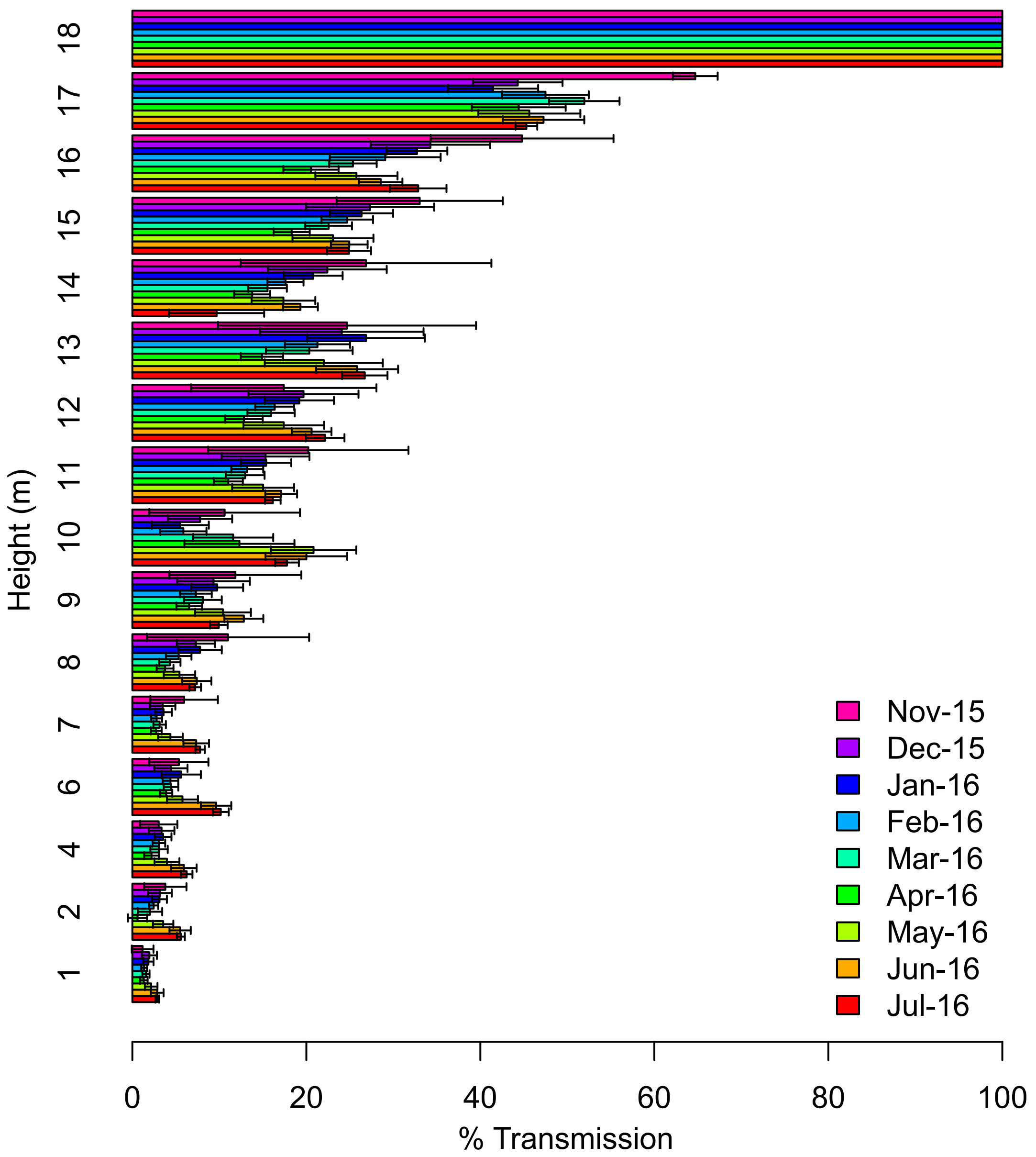

Figure S2. Light profile for sample secondary-2 determined monthly from continuous measurements between November 2015 and July 2016. Error bars show standard deviation between daily-determined % transmission for each height.

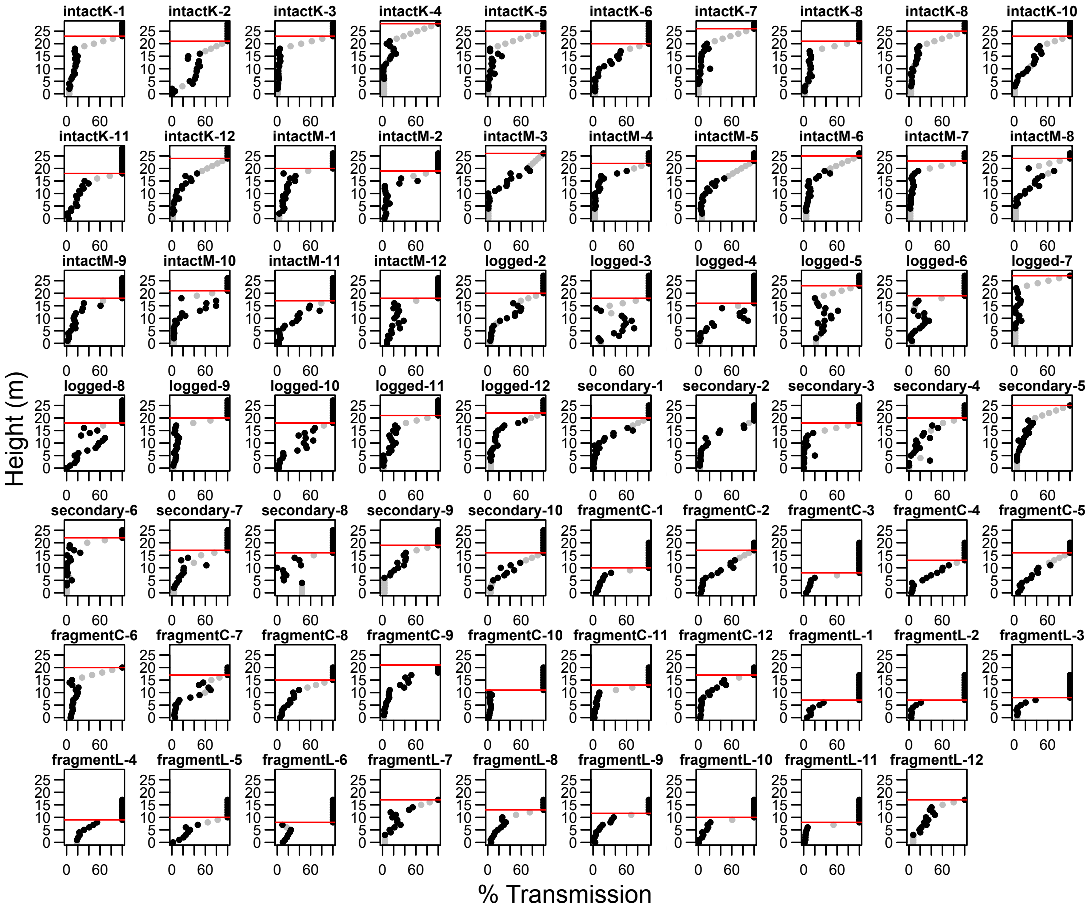

Figure S3. All light profiles, generated from PAR sensor data. Grey – interpolated or extrapolated data points. Red line – height at top of sample tree.

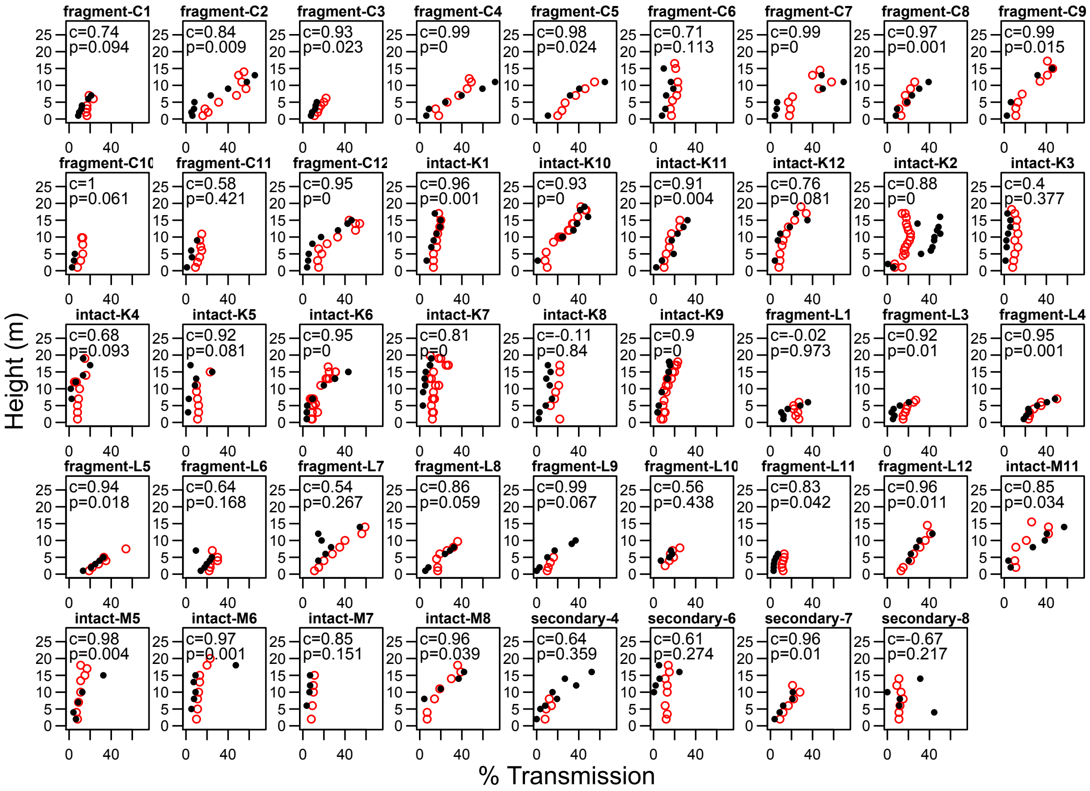

Figure S4. Light profiles from PAR sensors (black) and hemispherical photographs (red). Interpolated or extrapolated data points are not shown. c – Pearson correlation coefficient between sensor and photograph transmission, p – p-value of pearson correlation test.
